# Clinical Application Study of Polymeric Nanospheres Network in Methylphenidate Extraction from Urine Samples by Dispersive Solid Phase Extraction Adsorbent

**DOI:** 10.34172/apb.2022.054

**Published:** 2021-07-04

**Authors:** Arezou Taghvimi, Fatemeh Soghra Jahed, Siavoush Dastmalchi, Yousef Javadzadeh

**Affiliations:** ^1^Biotechnology Research Centre, Tabriz University of Medical Science, Tabriz, Iran.; ^2^Department of chemistry, Faculty of Science, Azarbijan Shahid Madani University, Tabriz, Iran.; ^3^Faculty of Pharmacy, Near East University, POBOX: 99138, Nicosia, North Cyprus, Mersin 10, Turkey.; ^4^Biotechnology Research Center, and Faculty of Pharmacy, Tabriz University of Medical Science, Tabriz, Iran.

**Keywords:** Polymeric nanosphere, Methylphenidate, Dispersive solid phase extraction, Urine

## Abstract

***Purpose:*** This research introduces a polymeric nanosphere as a new dispersive solid phase extraction (DSPE) adsorbent for the extraction of methylphenidate (MPH) from urine and its high performance liquid chromatography (HPLC) analysis.

***Methods:*** Polymeric nanosphere is a kind of copolymeric network obtained by copolymerization of an ionic liquid monomer and styrene in the presence of vinyltriethoxysilane and 2-hydroxyethylmethacrylate. HPLC coupled with ultra violet detector was applied for the determination and quantification of MPH. Dominant parameters in extraction were modified by the one-parameter-at-a-time method. The results are as follow: 10 mg of polymeric nanospheres (PNS), 400 μL of acetonitrile (ACT), 5 mL of urine with the pH value of 9, and the extraction and desorption times of 2 and 5 minutes, respectively, which can be selected as the optimum extraction conditions.

***Results:*** Calibration curve was plotted through optimized conditions, and the proposed method was validated. The results demonstrated that the method presented linearity in the concentration range of 30-1200 ng/mL. Selectivity, matrix effect and metabolites interference effect were investigated and the method presented no obvious interference effect during the analysis run time. Repeatability, limit of detection (LOD) and limit of quantification (LOQ) values of the method can be reported in this section as well. The method showed satisfactory results with 98.8% relative recovery in the analysis of positive urine samples.

***Conclusion:*** The findings convinced the applicability of the introduced method for DSPE and HPLC analysis of the positive urine samples in different laboratories.

## Introduction

**Figure 1:**
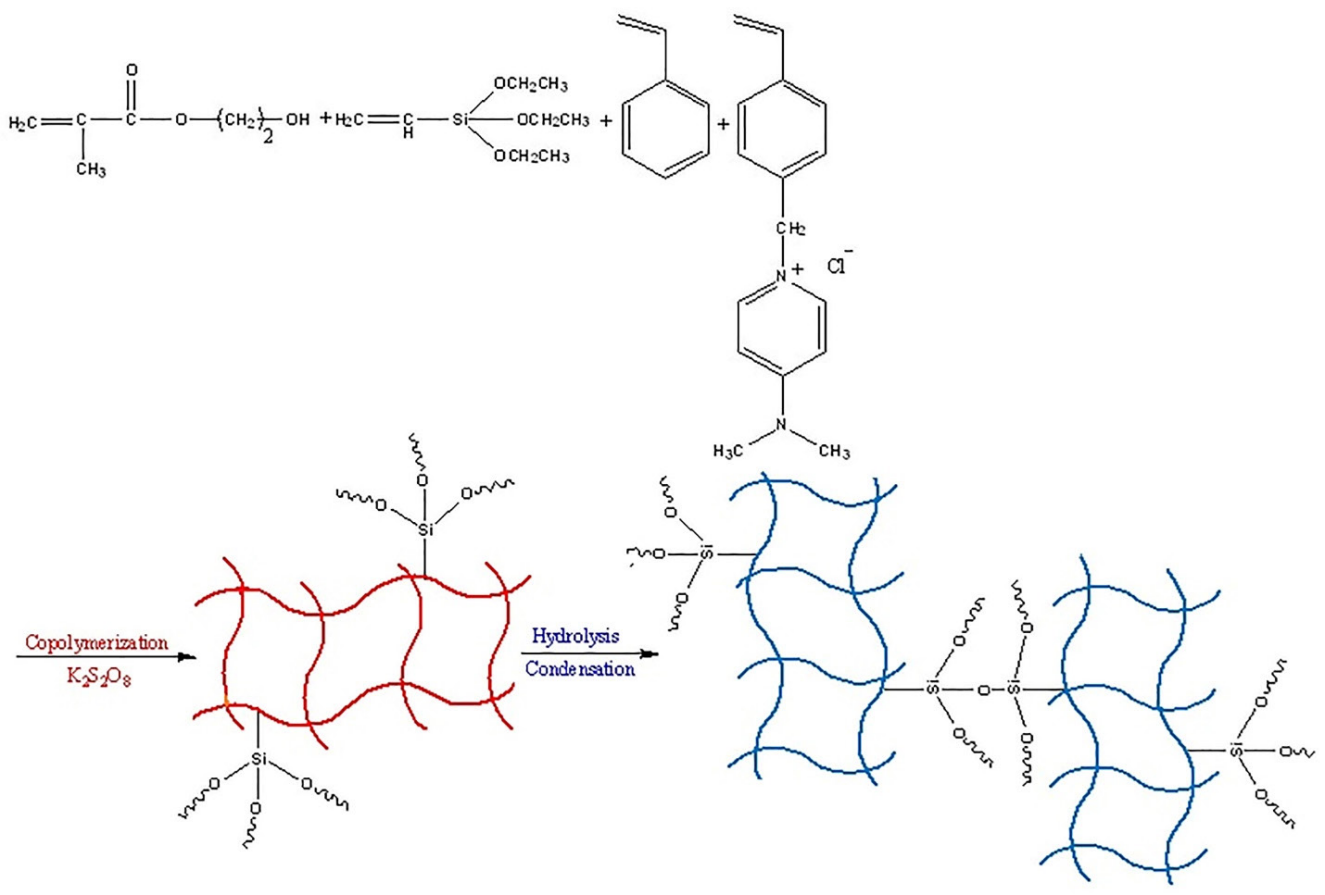
A Scheme of polymerization process of polymeric nanospheres.

Methylphenidate (MPH) is introduced as an effective therapy candidate for amphetamine-type abusers. Besides, MPH possesses an abuse potential and causes lots of social problems among young people.^1,2^ It is a psychostimulant drug classified as a phenethylamine derivative and is applied for narcolepsy and depression treatment.^3-5^ Attention deficit hyperactivity disorder is a controllable illness by MPH treatment especially in childhood.^6,7^ The supply of MPH in a treatment program is controlled, and distraction to illicit use is forbidden.^8,9^ Therefore, controlling the MPH level is very crucial in biological fluid in clinical and forensic laboratories.^1^ Biological fluids like plasma and urine are frequent matrices applied in most clinical laboratories.^10^ Besides, plasma is generally applied for MPH monitoring, but it might be a difficult sampling way in children and addicted subjects, thus urine and saliva are comfortable sampling techniques that are non-invasive.^11^ Sample preparation is an indispensable part of the analysis because of the complex matrix of biological samples and also low doses of analyte in the media.^12^ Solid phase extraction (SPE) is a well-known sample preparation step before analysis due to low organic solvent consumption in comparison to liquid-liquid extraction (LLE).^13^ Moreover, SPE is fast with the high preconcentration factor.^14^ SPE coupled with liquid chromatography–tandem mass spectrometry (LC–MS/MS) was applied to extract and determine amphetamine and MPH from wastewater samples.^15^ MPH was extracted and screened by derivatization and SPE steps coupled with gas chromatography mass spectrometry (GC-MS) in urine media.^4^ Automatic SPE and trimethylsilylation sample preparation method coupled with GC-MS was introduced for extraction and determination of MPH from humane urine.^16^ However,enantioseparation of MPH was carried out using a chiral fluorescent derivatization reagent in rat plasma.^17^ Electrophoretic derivatisation procedure was also mentioned using o-entafluorobenzyloxycarbonyl )-2,3,4,5- tetrafluorobenzoyl chloride for the quantitative determination of MPH in human plasma by use of LLE method.^18^ Miniaturized and effective form of SPE is called dispersive solid phase extraction (DSPE) method in which the adsorbent is directly in contact with the analyte solution resulting in high extraction efficiency.^19-21^ The lower amount of adsorbent is applied in the DSPE methods which is a highly considerable property.^19^ Polymeric composites are applicable in different research areas such as drug delivery, bioseparation, separation, and immunoassay.^22-24^ Large amounts of oxygen containing functional groups on polymeric surface area may provide the ability to design novel nano materials with highly developed physical and chemical properties. Modern synthesized ionic liquid (IL) based polymeric nanocomposite possessing hydrophilic groups like carboxylic acid on nanosphere surface area of nanoparticles provides well distributions of polymeric nanospheres (PNS) in the media and introduces novel DSPE adsorbent.^25,26^ This phenomenon increases the analyte interaction with the nanosphere surface and then elevates the extraction efficiency. Moreover, the selective extraction of MPH with positive charges from urine media is provided through the interaction of the MPH and the hydrophilic functional groups. PNS is a kind copolymeric network obtained by copolymerization of an ionic liquid monomer and styrene in the presence of vinyltriethoxysilane and 2-hydroxyethylmethacrylate. The polymerization condition brings to nanospheres. A scheme of the polymerization process is shown in Figure 1.

In the present paper, a novel polymeric nanosphere is synthesized, and after full characterization, it was used as a DSPE extraction adsorbent of MPH.

## Materials and Methods

### Chemicals and reagents

N, N-dimethylamino pyridine, 4-vinylbenzyl chloride, KH_2_PO_4,_ HCl, NaOH, methanol (MT), acetone (ACE), and acetonitrile (ACT) (in HPLC grade) were provided from Merck Chemicals (Darmstadt, Germany). 2-hydroxyethylmethacrylate, vinyltriethoxysilane, styrene, tetrahydrofuran, and potassium persulfate were purchased from Sigma Aldrich (USA). MPH hydrochloride stock solution and ritalinic acid stock solution 1000 µg/mL in MT were kindly donated by Mehrdarou pharmaceutical Co., (Karaj, Iran). All synthesis procedures were done under an inert atmosphere. Ultrapure water was prepared by Milli-Q water system (Darmstadt, Germany).

### Instrumental conditions

HPLC analysis was performed using Knauer (Germany) system. The analytical C_18_ column (5 µm particle diameter, 4.6 mm i.­ d. × 25 cm) (Knauer, Germany) was applied for separation. The mobile phase consists of ACT/phosphate buffer solution with the ratio of 20:80 (V/V) (10 mM, pH = 3.5), applied at the flow rate of 1 mL/min. A Fourier transform infrared (FT-IR) spectrometer (Tensor 27, Bruker, Germany) was applied in the range of 400-4000 cm^-1^ to characterize the synthesized PNS. Scanning electron microscope (SEM), MIRA3 FEG–SEM (Tescan, The Czech Republic) was used for the morphologic survey. Powder X-ray diffraction (XRD) was carried out by (D5000, Siemens, Germany) with a Cu tube anode.

### Method

#### Synthesis of polymer nanospheres

The IL monomer was synthesized by a procedure stated in previous work.^27^ More information is reported in S1. The N-(4-vinylbenzyl)-4-(N,N-dimethylamino) pyridinium chloride monomer (1 g) and water (70 mL) were added into a 250 mL three-necked flask with a magnetic stirrer, condenser, and an ampoule. The mixture was stirred at room temperature for 1 minutes to dissolve the IL monomer. Then a mixture of styrene (1.8 g), vinyltriethoxysilane (3.8 g), and 2-hydroxyethylmethacrylate (2 g) were added. After stirring at 75°C for 30 minutes under an argon atmosphere, potassium persulfate (0.2 g) dissolved in water (10 mL) was added via an ampoule. The temperature was raised to 80°C, and the reaction was preceded for 24 hours. Finally, the obtained precipitate was filtered and washed with deionized water three times. The resulting product was dried at 70°C for 24 hours.

#### Biological sample preparation

The optimization steps were carried out by mixing the urine samples of three healthy volunteers. The purpose was to decrease the effect of the urine matrix of various healthy subjects. The samples were kept at 4℃. Addicted subjects’ urine samples were provided from MAHAN therapy center (Tabriz, Iran). All the participants accepted to take part in the project and approved the ethical consent. Urine samples were kept just for 2 or 3 days and then they were applied in the project. Sample preparation was started by spiking 0.1 µg/mL MPH into 5 mL urine sample. The sample pH was alkalized by adding KOH 1M until the pH of the sample reached 9, leading to the precipitation of insoluble solid particles. The insoluble particles were separated by centrifugation at 5500 rpm for 10 minutes.

#### Extraction procedure

The extraction was processed by adding 10 mg of the PNS into 5 mL of 0.1 µg/mL MPH spiked sample. The sample was sonicated (30 seconds, Farasout, Iran) for 4 min to provide the maximum interaction of analyte and PNS. Then, it was centrifuged, and the supernatant was removed, and 400 µL of desorption solvent was added and sonicated for 5 minutes later. The resulted solution was centrifuged once more, and 20 µL of the obtained liquid was injected into the analytical HPLC instrument.

## Results and Discussion

### Characterization of polymeric nanospheres

**Figure 2:**
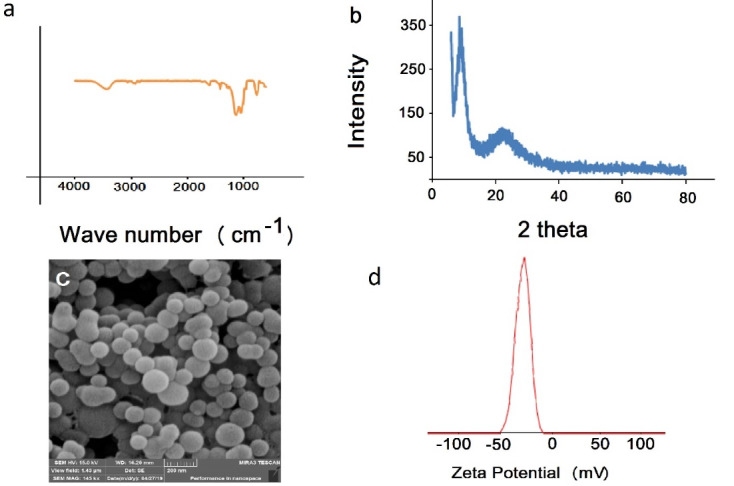
a) Present the FTIR of the PNS, b) XRD pattern of the synthesized PNS, c) SEM image of and d) zeta potential value.

The FTIR spectrum of synthesized polymer is shown in Figure 2a. The bands at 3066 cm^-1^ and 2853-2961 cm^-1^ are related to the stretching vibration of C-H aromatic and aliphatic bands, respectively. The band at 1739 cm^-1^ belongs to C = O band that confirms the presence of HEMA in the polymer structure. The aromatic peaks of C = C appeared at 1609 cm^-1^ and 1413 cm^-1^ that confirm the presence of styrene and pyridine aromatic rings. Observation of the stretching vibration of Si-O-Si at 1138 cm^-1^ confirms the hydrolysis and condensation of Si (OR)_3_ group in the polymer network. Figure 2b shows the XRD of synthesized polymer nanospheres. The diffraction peaks at 2Ɵ of 9° and 23° confirm that the polymer has a certain crystallinity. The SEM image of synthesized nanospheres is depicted in Figure 2c. The synthesis process leads to PNS with an average particle size of 100 nm. The polymeric nanocomposite SEM image presents smooth and uniform particles without any aggregation and amorphous phase particles. Figure 2d shows the zeta potential value of the PNS nanocomposite. The obtained -32.5 mV potential value supports the presence of the carboxylic acid group in the PNS structure.

### Extraction process

The extraction mechanism might be described as follows: the synthesized PNS possesses hydrophilic groups like carboxylic acid on the nanosphere surface area of nanoparticles that provide well distributions of PNS in the media. Moreover, negative charges on PNS nano-adsorbent served by carboxylic acid functional groups provide well interactions between the positively charged MPH and negatively charged PNS. The systematic one-factor-at-a-time experimental design was utilized for the optimization of influential extraction parameters. Parameters as ionic strength, volume, pH, the kind and amount of the extraction organic solvent, the amount of the adsorbent, and desorption and extraction times were examined.

#### Adsorbent amount

**Figure 3:**
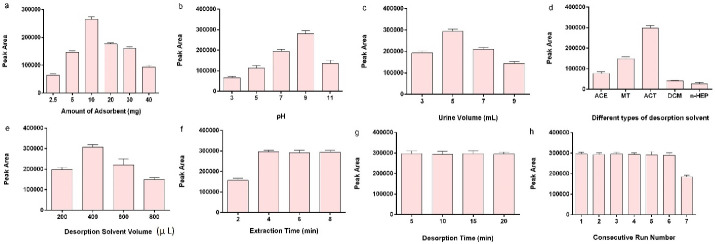
Optimization of the effective extraction parameters.

Adsorbent quantity influences the maximum interaction between the analyte and PNS by providing efficient interaction surface area. For this purpose, various amounts of the adsorbent in the range of 2.5-40 mg were studied. The results revealed that the extraction efficiency increased by the gradual increase of the adsorbent from 2.5 mg to 10 mg. The continuous increase of the adsorbent led to a decrease in the extraction peak area. Aggregation of the PNS network is a probable reason for the lower extraction efficiency (Figure 3a).

#### Urine pH, urine volume and ionic strength

The pH value can change the effective charges on the adsorbent and also the analyte, which may escalate or diminish the effective interaction between the MPH and PNS. The procedure would change the extraction efficiency. As a result, the pH of the sample was investigated in the range of 3-11. The amount of effective negative charges on the PNS decreases when the pH value is 3 due to highly positive ions in the urine media (Figure 3b). Therefore, the interaction between the PNS and positively charged analyte will deplete. When the pH increased to 5, the ionization of the carboxylic acid group increased. This may help to achieve high PNS dispersion in the environment and provide a suitable surface for the adsorption of the analyte. The steady increase of pH up to 7 contributed to the extraction efficiency due to the ionization of MPH, which in turn increased the interaction between the negative charge adsorbent and the positive charge analyte. When MPH is half-separated and partially ionized (pH 9), its benzene ring approaches the PNS and participates in the interaction. The two mechanisms of interactions improve the extraction peak area. Therefore, maximum extraction efficiency was achieved when the pH was adjusted to 9. In higher alkaline pH values (pH 11), there was no tendency to the highly negative surface charge of PNS and un-dissociated analyte; therefore, the extraction efficiency declined in pH 11. Different urine volumes of 3-9 mL were studied (Figure 3c). The results showed that the enhancement of the urine volume from 3 to 5 mL raised the dispersion of the PNS in the urine, increasing the effective analyte and PNS interaction. The expansion of the urine volume up to 9 mL reveals a depression in the peak areas. This phenomenon might be related to the dissociation of the nanoparticles and might inferior effective interactions. The ionic strength of the media was studied in 0-8 % (W/V) of NaCl as well. No apparent change was detected in the extraction efficiency. The experiment was carried on without ionic strength adjustment.

#### Desorption solvent and its volume

Desorption solvent provides well-matched interaction toward the adsorbed analyte and is capable of maximum desorption of the analyte from the PNS surface. This process changes the extraction capability. Therefore, different solvents like ACE, MT, ACT, dichloromethane (DCM), and n-heptane (n-HEP) were inspected (Figure 3d). The results demonstrated that ACT presented maximum extraction efficiency due to providing suitable interaction polarity between the adsorbent structure and the MPH. The volume of the ACT was modified in the range of 200-800 µL (Figure 3e). The data demonstrated that the greatest extraction efficiency was achieved in 400 µL of the ACT, and further addition of the ACT decreased the extraction efficiency due to analyte dilution.

#### Extraction and desorption sonication time

In the equilibrium methods, the length of time required for reaching the equilibrium is crucial. Therefore, it is a parameter that should be optimized. The extraction time was investigated by sonication at the time range of 2-8 minutes (Figure 3f). In the time range of 2-4 minutes, the extraction efficiency was enhanced, but more sonication time diminished the extraction efficiency. This process shows that the extraction was reaching the equilibrium in 4 minutes and more increasing of the sonication time is not effective. This survey was processed for desorption time in the time range of 5-20 minutes (Figure 3g); 5 minutes is enough for the establishment of the desorption equilibrium.

#### Reusability of the method and adsorbent capacity

Figure 3h shows the reusability of the PNS covering the optimized conditions. The outcomes demonstrated that no significant variation was observed in the peak area during six repetitions (*P* < 0.05). The adsorbent capacity for synthesized PNS was obtained according to the following equation:


$$Q_{e}=\left(\frac{\left(C_{0}-C_{e}\right)V}{M}\right)\times 100$$

C_0_ and C_e_ are the initial and equilibrium concentrations of MPH in the solution (µg/mL), respectively. V is the urine volume (mL), and M is the adsorbent amount (g). The Q_e_ was calculated as 2.04 mg/g.

### Method validation

Limit of detection (LOD), coefficient of determination (r^2^), relative standard deviation (RSD), the limit of quantification (LOQ), and linear range are some of the analytical characteristics for the developed method for MPH determination. The data obtained through the calibration curve under optimized conditions for five concentrations were repeated three times. The method is linear in the concentration range of 30-1200 ng/mL. LOD and LOQ of the method were calculated 11.0 and 28.40 ng/mL, respectively. Furthermore, RSD and the linearity of the method were found to be 6% and 0.9931, respectively. To completely study the accuracy and precision of the proposed method, analytical accuracy and precision were surveyed by examining six healthy subjects’ urine samples. The results are given in Table 1. The advantages of the proposed study over some other published papers are presented in Table 2. A higher recovery percentage was obtained in this study, and no derivatization step is required for the sample preparation step, which is usually time-consuming and easy. Moreover, toxic and unsafe chemicals might be used in the derivatization step that is not applied in the introduced method. A slightly higher detection limit obtained in this method is attributed to the instrumental limitation of the UV detector. The method for calculations of preconcentration factor, spike recovery and extraction efficiency are as bellow:


Preconcentrion factor=AUC spikeAUC STDExtraction Efficiency=Slope of calibration curve after exytractionSlope of calibration curve before exytraction*100Spike Recovery=AUC spikeAUC STD*100

**Table 1:** Analytical precision and accuracy

** Analyte concentration (µgmL^-1^) **	**Intra-day (n=3)**		**Inter-day (n=3)**	
	**Precision** **RSD (%)** ^a^	**Accuracy** **(bias)**	**Precision** **RSD (%)**	**Accuracy** **(bias)**
0.1	4.75	1.02	5.76	1.4
0.5	4.83	1.57	5.80	1.9
1.2	5.1	1.39	5.04	1.06

^a^Relative standard deviation.

**Table 2:** Some analytical characterizations and advantages of the proposed method over the methods reported in previous reports

**Method**	**Matrix**	**LOD**	** Linear Range (ngmL^-1^) **	**Recovery (%)**	**Disadvantage**	**References**
SPE-LC-MS-MS^a^	Waste water	0.0006	-	63.33	Low recovery	15
LLE-GC-NICI-MS^b^	Plasma	-	0.006-12.5	-	Derivatization step	18
LLE-HPLC-FL^c^	Plasma	1.0	1-80	65.72	Derivatization step, low recovery	28
DSPE-HPLC-UV^d^	Urine	11.0	30-1200	98.8	Slightly high detection limit	This Study

^a^Solid phase extraction liquid chromatography- tandem mass spectrometry, ^b^Liquid-liquid extraction-gas chromatography- negative ion chemical ionisation mass spectrometry, ^c^Liquid-liquid extraction-high performance liquid chromatography-fluorescence detector, ^d^Dispersive solid phase extraction- high performance liquid chromatography-ultra violet detector.

#### Analysis of real samples

**Figure 4:**
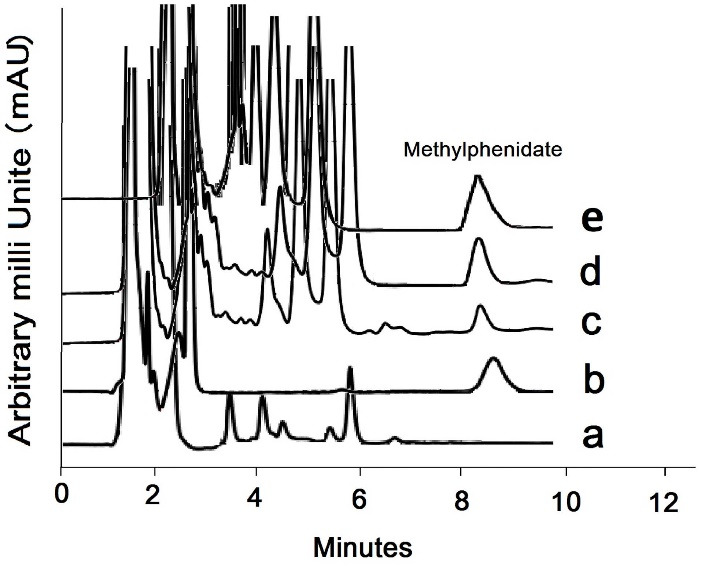
(a) Chromatograms of the blank and (b) spiked urine sample with 0.1 µg/mL methylphenidate, (c) chromatograms of addicted subject and (d) selectivity study of the proposed method the presence of 0.1 µg/mL amphetamine, ritalinic acid, ephedrine and pseudoephedrine in urine media, (e) Chromatogram related to the addicted subjects’ urine spiked with 0.1 µg/mL MPH.

Blank and 0.1 µg/mL MPH spiked urine sample chromatograms are displayed in Figure 4a and 4b, respectively. Addicted subjects’ samples were analyzed using the introduced method (Figure 4c). The obtained relative recovery was 98.8%. The average amount of MPH was found to be 1.38 µg/mL in real urine samples. The chromatogram related to the addicted subjects’ urine spiked with 0.1 µg/mL MPH is shown in Figure 4e.

#### Selectivity and matrix effect

The selectivity of the method was carried out by simultaneous spiking of 0.1 µg/mL MPH metabolite such as amphetamine, ritalinic acid, and some other drugs like ephedrine and pseudoephedrine in urine media, and the extraction process was carried out under optimized conditions. According to Figure 4d, no meaningful peak related to the ritalinic acid or other interfering drugs during the analysis run time. was seen during run time. The matrix effect was carried out by evaluating the level of enhancement or reducing of MPH peak area made by the urine matrix as follow:

Matrix Effect (%) = B/A ×100

A is the peak area of the standard solution, and B is the peak area of post-extracted sample solution. The matrix effect was 101%, confirming a minor matrix effect.^29^

## Conclusion

The current study presented a novel established sample preparation method for extraction of MPH from urine media. The synthesized new PNS were applied for the DSPE of MPH for the first time. The synthesis process of PNS adsorbent is comfortable and safe. Furthermore, the sample preparation step is fast and cost-effective. The matrix effect studies showed no significant enhancement or reduction of MPH peak area made by the urine matrix. Satisfactory outcomes from real urine sample analysis confirmed the applicability of the proposed method in various clinical laboratories.

## Acknowledgments

The researchers kindly appreciate the National Institute for Medical Research Development (NIMAD) for financial supports of this study (Grant number: 971219).

## Ethical Issues

All procedures performed in studies involving human participants were in accordance with the ethical standards of the institutional and/or national research committee and with the 1964 Helsinki declaration and its later amendments or comparable ethical standards.

## Conﬂict of Interest

No conﬂict of interest.

## References

[1] Paterson SM, Moore GA, Florkowski CM, George PM (2012). Determination of methylphenidate and its metabolite ritalinic acid in urine by liquid chromatography/tandem mass spectrometry. J Chromatogr B Analyt Technol Biomed Life Sci.

[2] Solanto MV (1998). Neuropsychopharmacological mechanisms of stimulant drug action in attention-deficit hyperactivity disorder: a review and integration. Behav Brain Res.

[3] Cantwell DP (1996). Attention deficit disorder: a review of the past 10 years. J Am Acad Child Adolesc Psychiatry.

[4] Eichhorst J, Etter M, Lepage J, Lehotay DC (2004). Urinary screening for methylphenidate (Ritalin) abuse: a comparison of liquid chromatography-tandem mass spectrometry, gas chromatography-mass spectrometry, and immunoassay methods. Clin Biochem.

[5] Marchei E, Muñoz JA, García-Algar O, Pellegrini M, Vall O, Zuccaro P (2008). Development and validation of a liquid chromatography-mass spectrometry assay for hair analysis of methylphenidate. Forensic Sci Int.

[6] Shader RI, Harmatz JS, Oesterheld JR, Parmelee DX, Sallee FR, Greenblatt DJ (1999). Population pharmacokinetics of methylphenidate in children with attention-deficit hyperactivity disorder. J Clin Pharmacol.

[7] Peles E, Schreiber S, Linzy S, Domani Y, Adelson M (2015). Differences in methylphenidate abuse rates among methadone maintenance treatment patients in two clinics. J Subst Abuse Treat.

[8] Rösler M, Casas M, Konofal E, Buitelaar J (2010). Attention deficit hyperactivity disorder in adults. World J Biol Psychiatry.

[9] Capp PK, Pearl PL, Conlon C (2005). Methylphenidate HCl: therapy for attention deficit hyperactivity disorder. Expert Rev Neurother.

[10] Cruz-Vera M, Lucena R, Cárdenas S, Valcárcel M (2009). Sorptive microextraction for liquid-chromatographic determination of drugs in urine. TrAC Trends Anal Chem.

[11] Marchei E, Farré M, Pardo R, Garcia-Algar O, Pellegrini M, Pacifici R (2010). Correlation between methylphenidate and ritalinic acid concentrations in oral fluid and plasma. Clin Chem.

[12] Armenta S, Garrigues S, de la Guardia M (2008). Green analytical chemistry. TrAC Trends Anal Chem.

[13] Płotka-Wasylka J, Szczepańska N, de la Guardia M, Namieśnik J (2015). Miniaturized solid-phase extraction techniques. TrAC Trends Anal Chem.

[14] Eisert R, Pawliszyn J (1997). New trends in solid-phase microextraction. Crit Rev Anal Chem.

[15] Burgard DA, Fuller R, Becker B, Ferrell R, Dinglasan-Panlilio MJ (2013). Potential trends in attention deficit hyperactivity disorder (ADHD) drug use on a college campus: wastewater analysis of amphetamine and ritalinic acid. Sci Total Environ.

[16] Cheong JC, Suh SI, Ko BJ, Kim JY, In MK, Cheong WJ (2010). Gas chromatography-mass spectrometric method for the screening and quantification of illicit drugs and their metabolites in human urine using solid-phase extraction and trimethylsilyl derivatization. J Sep Sci.

[17] Inagaki S, Taniguchi S, Hirashima H, Higashi T, Min JZ, Kikura-Hanajiri R (2010). HPLC enantioseparation of α,α-diphenyl-2-pyrrolidinemethanol and methylphenidate using a chiral fluorescent derivatization reagent and its application to the analysis of rat plasma. J Sep Sci.

[18] Leis HJ, Windischhofer W (2011). Gas chromatography-negative ion chemical ionisation mass spectrometry using o-(pentafluorobenzyloxycarbonyl)-2,3,4,5-tetrafluorobenzoyl derivatives for the quantitative determination of methylphenidate in human plasma. J Chromatogr B Analyt Technol Biomed Life Sci.

[19] Rocío-Bautista P, González-Hernández P, Pino V, Pasán J, Afonso AM (2017). Metal-organic frameworks as novel sorbents in dispersive-based microextraction approaches. TrAC Trends Anal Chem.

[20] Wen Y, Chen L, Li J, Liu D, Chen L (2014). Recent advances in solid-phase sorbents for sample preparation prior to chromatographic analysis. TrAC Trends Anal Chem.

[21] Taghvimi A, Bavili Tabrizi A, Dastmalchi S, Javadzadeh Y (2019). Metal organic framework based carbon porous as an efficient dispersive solid phase extraction adsorbent for analysis of methamphetamine from urine matrix. J Chromatogr B.

[22] Bagheri H, Roostaie A, Baktash MY (2014). A chitosan-polypyrrole magnetic nanocomposite as μ-sorbent for isolation of naproxen. Anal Chim Acta.

[23] Chang YC, Chen DH (2005). Preparation and adsorption properties of monodisperse chitosan-bound Fe3O4 magnetic nanoparticles for removal of Cu(II) ions. J Colloid Interface Sci.

[24] Taghvimi A, Ghorbani M, Hamishehkar H (2018). Synthesis of a novel polymeric magnetic solid phase extraction adsorbent for selective extraction of amphetamine from urine samples coupled with high performance liquid chromatography. Drug Test Anal.

[25] He H, Yuan D, Gao Z, Xiao D, He H, Dai H (2014). Mixed hemimicelles solid-phase extraction based on ionic liquid-coated Fe3O4/SiO2 nanoparticles for the determination of flavonoids in bio-matrix samples coupled with high performance liquid chromatography. J Chromatogr A.

[26] Taghvimi A, Hamidi S, Javadzadeh Y (2019). Mixed hemimicelle magnetic dispersive solid-phase extraction using carbon-coated magnetic nanoparticles for the determination of tramadol in urine samples. J Sep Sci.

[27] Hosseinzadeh F, Mahkam M, Galehassadi M (2012). Synthesis and characterization of ionic liquid functionalized polymers for drug delivery of an anti-inflammatory drug. Des Monomers Polym.

[28] Zhu HJ, Wang JS, Patrick KS, Donovan JL, DeVane CL, Markowitz JS (2007). A novel HPLC fluorescence method for the quantification of methylphenidate in human plasma. J Chromatogr B Analyt Technol Biomed Life Sci.

[29] Van De Steene JC, Lambert WE (2008). Comparison of matrix effects in HPLC-MS/MS and UPLC-MS/MS analysis of nine basic pharmaceuticals in surface waters. J Am Soc Mass Spectrom.

